# The Incidence of Diagnosis of Munchausen Syndrome, Other Factitious Disorders, and Malingering

**DOI:** 10.1155/2019/3891809

**Published:** 2019-03-03

**Authors:** Harald Schrader, Thomas Bøhmer, Jan Aasly

**Affiliations:** ^1^Department of Neuroscience, Faculty of Medicine, Norwegian University of Science and Technology (NTNU), Trondheim, Norway; ^2^Department of Neurology, St. Olav's Hospital, University Hospital of Trondheim, Trondheim, Norway; ^3^Oslo University Hospital, Department of Medical Biochemistry, Faculty of Medicine, University of Oslo, Aker, Oslo, Norway

## Abstract

**Background:**

Systematic studies on factitious disorders and malingering in large populations are rare. To address this issue, we performed a nationwide epidemiological study in Norway on the incidence of these diagnoses in an unselected patient population. In particular, we tried to confirm the diagnoses and to estimate the contribution of Munchausen syndrome to the spectrum of factitious disorders.

**Methods:**

We analyzed data obtained from the Norwegian Patient Registry (NPR), which provided a deidentified list of all patients from 2008 to 2016 who had received the ICD-10 diagnosis of F68.1 or the diagnosis code Z76.5.

**Results:**

Altogether, 237 patients (99 females; 138 males) received a diagnosis of F68.1. Code Z76.5 was applied to 52 patients (12 females; 40 males), all diagnosed within health institutions. Three of 1700 specialists (somatic specialist, psychologist, or psychiatrist) in private practice had diagnosed a factitious disorder in altogether 87 patients. After contacting these specialists, we could identify no true case of F68.1. For 24 of 146 patients who were equally distributed by gender within health institutions, we managed to identify the diagnosing healthcare providers. Of these 24 patients, only 11 correctly qualified for code F68.1. Only two female patients qualified for a Munchausen syndrome diagnosis.

**Conclusions:**

There is a male predominance for the diagnosis of malingering. An earlier suspicion of a female predominance for Munchausen syndrome is upheld. There is significant underdiagnosing and misdiagnosing for both conditions and for factitious disorders in general. To separate the most serious form of factitious disorders from milder forms and to facilitate more systematic research, we recommend a specific ICD diagnosis for Munchausen syndrome.

## 1. Introduction

Despite great interest of both professionals and laypersons in the issue, there is a startling rarity of systematic research on factitious disorders, including Munchausen syndrome in particular, although it is now 68 years since the British physician Richard Asher described this peculiar disorder [1]. Munchausen syndrome as the most serious form of factitious disorder is included in the United States 2018 ICD-10 and specified by one of the 4 codes below F68.1 (factitious disorder) [2]. Epidemiological studies on the prevalence and incidence of diagnosis in the general population as well as controlled therapeutic or interventional studies are almost completely missing. More than 1200 publications on Munchausen syndrome are listed in PubMed, mostly related to clinical presentations and not to systematic studies. In addition, little systematic research in greater patient populations has been done on milder forms of the factitious disorder and on malingering.

To address this issue, we launched the first nationwide epidemiological study on the frequency of the diagnoses of factitious disorders and malingering given both outside and inside Norwegian health institutions. Through registration of all the ICD diagnoses in a central Norwegian Patient Registry (NPR), we could track the patients on their way through the health system and, by identifying several diagnosing health professionals, gather information hitherto not available.

We could find only a few previous studies on the incidence of confirmed diagnosis of factitious disorders, including Munchausen syndrome, done by experienced physicians [3, 4, 5]. One of them was submitted from a neurological department of one hospital in Berlin. During a period of one year, they detected that 4 out of 5 patients with a factitious disorder out of altogether 1538 patients (0.26%) presented the classical variant of Munchausen syndrome [3]. In another study, a team of experienced Italian medical professionals over a period of 2 ½ years found that 3 out of 751 children (0.4%) referred to a department of pediatrics in Rome fulfilled the criteria for Munchausen syndrome [4]. Over a period of 15 years, physicians from the Department of Internal Medicine at the University Hospital in Essen, Germany, observed 44 cases of self-induced factitious disorders, of which 7 were malingering and one was Munchausen syndrome, in this department alone [5]. Since the late sixties, the authors of the present study, during their employments at the National Hospital in Oslo and/or the university hospitals in Trondheim or Tromsø, diagnosed 8 patients with classical Munchausen syndrome, all of whom were females. Despite numerous admissions and contacts with other health institutions, none of these patients had previously received the same diagnosis.

In a National Hospital Discharge Survey on Somatoform, Factitious, and Related Diagnoses from abstracts of medical discharges submitted from a US nationwide sample of 400 to 500 general medical, short-stay, inpatient hospitals, an assignment rate of 6.8 per 100,000 (0.0068%) for factitious disorder was found [6]. The design of their study did not allow for confirmation of correctness of the diagnoses by the researchers themselves nor identification and confirmation of true cases of Munchausen syndrome.

By comparing the results of these studies with those of the present nationwide study and our own experience, we could evaluate the general awareness of healthcare providers concerning factitious disorders.

Our original focus was on the incidence of diagnosis of Munchausen syndrome, the most serious form of factitious disorder. However, no specific code for this condition exists, which therefore by use of code F68.1 is included in all other forms of factitious disorder. Therefore, we had to estimate the general incidence of diagnosis of F68.1 in the first place and then try to find ways by which we could estimate the contribution of Munchausen syndrome to the spectrum of the factitious disorder.

Malingering with the diagnosis code Z76.5 is the most important differential diagnosis to F68.1. By including this diagnosis in our study, we could determine the incidence of diagnosis of simulation in general.

## 2. Methods

We analyzed data from the Norwegian Patient Registry (NPR), a national institute that provides data for researchers and others seeking access to patient information. Data needed for the present study were available from 2008.

Following ethical approval by the Regional Committee for Medical and Health Research Ethics, the registry provided a deidentified list of all patients who, in the nine years from 2008 to 2016, had received the ICD-10 diagnosis of F68.1 or the diagnosis code Z76.5, which stands for “malingerer (conscious simulation).” The Norwegian version of the ICD-10, like the English version, defines F68.1 as “intentional production or feigning of symptoms or disabilities, either physical or psychological.” Furthermore, it provides the following description: “The patient feigns symptoms repeatedly for no obvious reason and may even inflict self-harm in order to produce symptoms or signs. The motivation is obscure and presumably internal with the aim of adopting the sick role. The disorder is often combined with marked disorders of personality and relationships.” F68.1 includes “Munchausen syndrome,” “doctor shopper,” “hospital hopper-syndrome,” and “peregrinating patient.” It excludes simulation with a specific motive, i.e., Z76.5.

The information supplied by the NPR contained a running number for tracking of each patient, year of birth, gender, year of contact with a healthcare provider, the name of the health institution, if it was within a somatic or psychiatric/psychological sector, and the name of one of the approximately 1700 specialists (somatic specialist, psychologist, or psychiatrist) who have an operation agreement with one of the four Norwegian Regional Health authorities. Operation agreement means that the specialists, in return for financial support from the health authorities, have several obligations, such as specified opening hours of their private practice and reporting of ICD diagnoses to the National Patient Registry. In 2016, the operation agreement specialists evaluated and treated 25 percent of all outpatients. Health institutions provided the remainder of the data relating to the outpatients and all the inpatient data, although the patient data from health institutions did not contain any information about whether the diagnoses were made in out- or inpatients.

For the confirmation of the diagnoses, we were limited to contacting healthcare providers who could remember their case(s) and who were able to correctly identify the patient(s) by the ICD diagnosis of F68.1 they had sent to the NPR. The original diagnosing healthcare provider could, in case of any uncertainty, reread the original patient journal for final control and for elimination of any recall bias. This approach limited the number of confirmed diagnoses, these mainly being provided by specialists whose names appeared in the NPR data and who could be easily contacted. The heads of the departments within health institutions that we could identify and that identified the patients in question received a letter informing them about the diagnostic criteria of F68.1 (especially Munchausen syndrome), Z76.5, and somatization disorder, the latter two conditions being the most relevant differential diagnoses to F68.1. We asked whether the patient qualified for the F68.1 diagnosis as described by the symptomatology according to the criteria of the United States 2017/18 ICD-10-CM version of Diagnosis Code F68.1 effective on October 1, 2017 [2]. We attached an empty table in which the healthcare provider could put a cross on the category that matched, and we indicated that we did not need any detailed clinical information about the patient who therefore remained entirely anonymous to us. The healthcare provider who made the diagnosis in the first place could, alternatively to making a cross on the table, inform us about the corrected/adjusted category or a diagnosis completely different to the proposed ones by telephone or in an ordinary email text. For a better understanding of the differential diagnoses, especially malingering and somatization disorder, we added a link to a previous article we had published in a Norwegian medical journal about challenges presented by Munchausen syndrome [7].

If a diagnosis of F68.1 was confirmed, the provider was asked whether the patient conformed to the classical clinical picture of Munchausen syndrome as described first by Asher in 1951 [1] and extended by Ireland et al. in 1967 [8]. This includes feigned severe illness of a dramatic and emergency nature; factitious evidence of disease, surreptitiously produced by interference with diagnostic procedures or by self-mutilation; a history of many hospitalizations; extensive travel or visits to innumerable physicians; evidence of laparotomy scars or burr holes; pathologic lying; aggressive, unruly, evasive behavior; and departure from the hospital against medical advice.

In all cases with confirmed F68.1 diagnosis, having classical Munchausen syndrome or not, the physician or psychologist informed us whether the case was considered an unspecified factitious disorder (F68.10) or a factitious disorder with predominantly psychological signs and symptoms (F68.11), with predominantly physical signs and symptoms (F68.12), or with combined psychological and physical signs and symptoms (F68.13).

If the patient did not qualify for F68.1, we asked for another diagnosis, for example, Z76.5 (malingerer/conscious simulation/), F45.0 (somatization disorder), or other diagnoses.

In patients with the diagnosis code Z76.5, we employed the same methodology, contacting the identifiable healthcare provider for either confirmation or change of the original diagnostic code.

## 3. Results

A total of 237 patients (99 females and 138 males) with an average age of 38 years were given the diagnosis of F68.1, making an average annual incidence of approximately 5 patients per million Norwegian inhabitants (Table 1). The diagnosis code Z76.5 was given to 52 patients, predominantly men (12 females and 40 males). These were all diagnosed within health institutions. An average annual incidence of the condition of approximately one patient per million Norwegian inhabitants became apparent. The numbers and percentages of the patients diagnosed with F68.1 in the different disciplines are shown in Table 2, the greatest contributor being within the psychiatric/psychological sector.

In 386 contacts (41%), F68.1 was the principal code, and in 551 contacts (59%), the principal code was another diagnosis. The corresponding numbers for the diagnosis code Z76.5 were 37 (52%) and 34 (48%).

Just three specialists with an operation agreement had diagnosed a factitious disorder in altogether 87 patients. After contacting these specialists and informing them of the diagnostic criteria, no true case of F68.1 was identified. In 86 cases (26 females and 60 males diagnosed by one neuropsychologist and one female diagnosed by a specialist in neurology), the correct diagnosis code should have been Z76.5, as there was a motive to simulate because they were claiming monetary compensation (*n* = 85) or benefits (*n* = 1) from the Norwegian National Health Service. In the first set of cases mentioned, simulation was detected through their cognitive underperforming in neuropsychological testing in the context of litigation after minor head and/or neck injuries. One female patient, diagnosed by a psychiatrist, probably had a somatization disorder. Four male patients, incarcerated persons, were far more likely to qualify for Z76.5. The remaining 146 patients had an average number of admissions and/or consultations of 5.7 (range: 1-94). Fifty percent were females.

**Figure 1:**
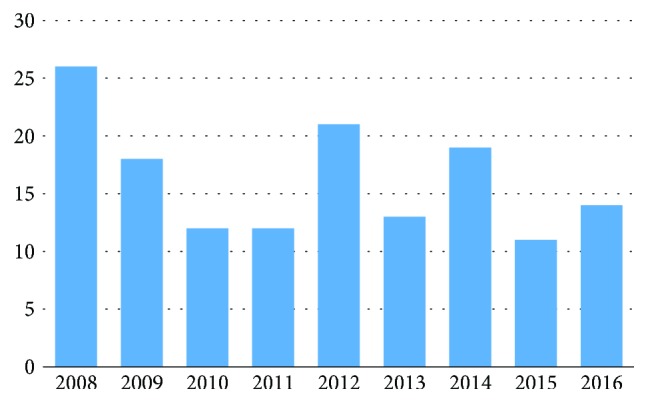
Frequency of ICD-10 diagnosis code F68.1 per year from 2008 to 2016 in 146 patients.

There was no significant trend of the frequency of F68.1 diagnosis in the 146 patients in the years 2008 to 2016 (Figure 1). The average number of institutions the 146 patients had received their diagnosis from was 1.18.

Of these patients diagnosed in health institutions, we could confirm only 24 cases provided by healthcare providers who had coded them as F68.1, mainly between the years 2013 and 2016, or remembered the patients individually because of frequent hospitalizations or consultations (Table 3). The vast majority of cases (*n* = 19) came from smaller health institutions. For larger hospitals, it was, with few exceptions (*n* = 5), an almost unsurmountable task to identify the diagnosing physician. Fortunately, each department that we could identify as the place where a diagnosis of F68.1 was given had very few patients with this diagnosis. If the head of the department did not clearly remember the patient in question based on the information about age, gender, and year of assignment of the diagnostic code, then the diagnosing physician in most cases knew the patient very well. Cases for which there was any degree of uncertainty about the patient (for example, because the diagnosing physician no longer worked in the health institution or the original electronic patient journal was not detailed enough) were not included in the subsample.

**Figure 2:**
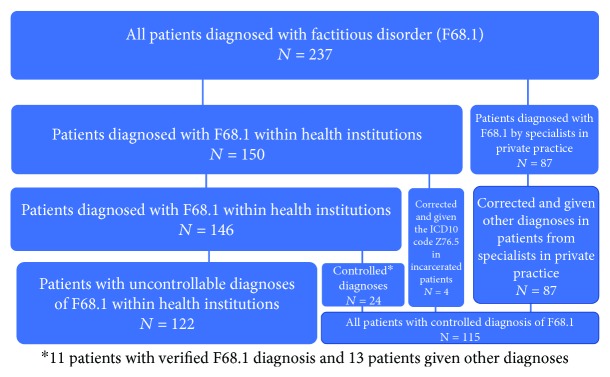
Flowchart showing, of the original patients who were assigned to a factitious disorder between 2008 and 2016 in the Norwegian Patient Registry, for how many the diagnosis could be controlled and eventually corrected. Of the original 237 patients, 122 patients within health institutions remained with uncontrollable diagnoses.

Of the 24 patients, 11 patients had a true factitious disorder and only two female patients qualified for the diagnosis of typical Munchausen syndrome by having most of the characteristics. The other 9 patients with a true factitious disorder in the subsample were far from having Munchausen syndrome. In the whole subsample, the diagnosis was given an average of 17.5 times, whereas the remaining 122 patients (Figure 2) received the diagnosis 3.5 times on average. In 81 (66%) of these 122 cases, the diagnosis was given only once, and in only three cases, 17 or more times. The patients not included in the subsample were, therefore, far less likely to have Munchausen syndrome. Consequently, in order to estimate the number of patients with Munchausen syndrome in the whole sample, it did not seem meaningful to use statistical methods such as calculating the confidence intervals. We were instead limited to extrapolating the two patients with confirmed diagnosis in the subsample to the 146 cases with F68.1, assuming that this would probably represent the maximum. The resulting number was 12 patients, corresponding to a presumed maximal annual incidence of 0.27 diagnosed patients with Munchausen syndrome per million inhabitants. In the NPR, over the nine years examined, altogether 5,177,984 patients were registered with an ICD diagnosis, meaning that a maximum of 0.00023% were diagnosed with Munchausen syndrome.

When summing the patients who retrospectively were diagnosed as Z76.5 with those who had the diagnosis in the first place, a total of 144 patients (52 + 86 + 6: 39 females and 105 males) had the diagnosis of malingering. The diagnosis could only be confirmed in four of the 52 patients with the original code Z76.5. In two cases it should have been F68.1. When subtracting the two cases that should have been F68.1, a maximum of 142 patients qualified for the diagnosis of malingering.

## 4. Discussion

With a maximum of only 0.00023% of all registered patients having received a diagnosis of Munchausen syndrome and a maximum of 135 patients (0.0026% of all registered patients, equally distributed by gender) having received the correct diagnosis of factitious disorder with all grades of severity, our national study, in comparison with small previous studies and our own experience, supports the impression of significant underdiagnosis. The latter rate was lower than that found in the National Hospital Discharge Survey. This is probably due to the fact that our diagnostic rate represents individual patients irrespective of the number of assignments, whereas the study by Hamilton et al. [6] generated data that represents hospitalizations and not patients.

As in Hamilton et al.'s study [6], the lack of confirmation was a severe limitation of the present study. Nevertheless, we managed to control all F68.1 codes given by specialists with an operation agreement and in 24 of 146 cases from health institutions. The latter was a challenging and time-consuming task which succeeded only because of the relative transparency of the Norwegian health system. Additionally, many healthcare providers cooperated sympathetically because they had already read our previous article about the challenges of Munchausen syndrome in the Norwegian medical journal [7] or read it with the help of a link in the letter they received from us. Recall bias was avoided by only including patients who were unequivocally identified by the original diagnosing healthcare providers and their access to a detailed electronic patient journal.

There are various reasons for the underdiagnosis of Munchausen syndrome. First, most patients will flee the ward when confronted with a distrustful physician. The few patients with whom contact is maintained usually refuse to participate in evaluation and seldom consent to psychiatric evaluation and treatment. The clinical presentation is diverse with a large spectrum of simulated conditions and falsified findings. Munchausen syndrome is the most severe form of factitious disorder, and the patients expose themselves to life-threatening surgical procedures, invasive examinations, and unnecessary treatments. However, the disorder lacks its own code that would separate it from milder forms. Additional important factors may be a low awareness of the condition and that physicians generally are reluctant to identify simulating patients. Even if they manage to identify simulation, out of fear of stigmatizing the patient, they often hesitate to put the correct ICD-10 diagnosis on top of the discharge summary sent to the referring physician and other earlier involved health institutions.

In the present study, in addition to underdiagnosis, we also detected many cases of misdiagnosis, most often attributed to confusion regarding the difference between ICD-10 diagnosis codes F68.1 and Z76.5. Out of 237 patients, coded as F68.1 by Norwegian healthcare providers between the years 2008 and 2016, the diagnosis was correct in fewer than 133 of patients within health institutions. All diagnoses made by private specialists were incorrect.

Misdiagnosis of the simulating patient even in published cases is not seldom. This is shown by a study of case reports and case series of adult patients with factitious disorders (FD) in the MEDLINE database, where 11.3% did not meet DSM-5 (Diagnostic and Statistical Manual of Mental Disorders-5) diagnostic criteria [9].

As for patients from health institutions, we could confirm the diagnosis in only a subsample of 24 patients that the diagnosing physician or psychologist remembered and clearly identified. We found that in 11 cases, the F68.1 diagnosis could be upheld, but only two of them had a relatively typical Munchausen syndrome. Both were females in accordance with the female preponderance observed in our own experience and the results of a systematic review of 455 cases in the professional literature [10]. Extrapolating these two cases to the maximum of 146 patients with F68.1, altogether a presumed maximum of 12 patients with a diagnosis of this severe form of factitious disorder emerged. The true total number of patients having a Munchausen syndrome and whom Norwegian healthcare providers identified in these nine years is probably even lower.

As for code Z76.5, specialists with an operation agreement outside institutions diagnosed no cases, but after instruction, one neuropsychologist and one physician corrected all their original F68.1 diagnoses to this diagnosis code. On questioning the healthcare providers, the reason appeared to be uncertainty regarding the diagnostic criteria and a reluctance to use the more offending wording “malingerer” in contrast to the milder wording according to the definition of F68.1 in the Norwegian ICD-10.

In Norway, in 2008, there were 180 registered specialists in neuropsychology, most of them employed in health institutions and only a few in private practice. Our impression is that few of them use validated tests to determine aggravation or simulation of cognitive deficits and/or give it the proper weight in their expert testimony. This is despite the fact that by using such tests it has been shown that many litigants probably are malingering [11].

The results of the present study confirmed the above suspicion. One neuropsychologist, although regularly erroneously making the diagnosis of F68.1, examined 85 patients with the diagnosis of simulation. She, in contrast to many other neuropsychologists, had incorporated validated simulation tests in her neuropsychological test battery and reported the diagnosis to the NPR. If all the other neuropsychologists engaged in litigation in Norway had done the same and made sure that the correct ICD-10 diagnosis code of Z76.5 was conveyed to the NPR, the annual incidence of diagnosis of malingering in the present study would have increased by an order of magnitude.

In Norway, every inhabitant has a unique personal identification number (PIN), and there is a national registry for ICD diagnosis. This makes it possible to track patients on their way through the health system. It also provides a good window of opportunity for identification and high transparency when a comprehensive discharge summary with the diagnosis F68.1 is sent to other involved health institutions. It would provide the opportunity in Norway and other countries with a similar registration system to perform much-needed controlled studies by which one could test interventions that could motivate patients to stop their self-harming behavior.

Treating patients with Munchausen syndrome appears to be difficult. For the very few patients who can be motivated to engage in psychotherapy and/or medicinal treatment, the success rate is very low. The health system is therefore subjected to a burden, which even for a single patient may be large, with much greater resources spent than on patients with organic conditions. We cautiously estimate that the total costs for each of our 8 patients have averaged more than 105,000 Euros [7].

There may be solutions. We have followed our eight patients for several years (in one case for 15 years) and have been able to demonstrate that sending a detailed discharge summary, not only to the primary doctor and referring doctor or health institution but to all health enterprises that the patient has been in contact with according to their own verified information, has resulted in the person concerned either stopping their behavior or having their activity considerably reduced.

In our clinical experience, other practitioners seldom use discharge summaries in making the diagnosis F68.1 or send them to health institutions. This assumption is supported by the fact that, on average, only slightly more than one institution had made the F68.1 diagnosis per individual patient. The reason for not distributing discharge summaries with the diagnosis of a factitious disorder like Munchausen syndrome seems to be that many health professionals feel uncomfortable about this approach, feeling that they may stigmatize the patient. However, in our view, attempting to protect the patient against serious self-inflicted and iatrogenic injuries, which sometimes put their lives at risk, is an ethically higher objective than simply accepting their behavior.

## 5. Conclusions

There is a male predominance for the diagnosis of malingering and the suspicion of a female predominance concerning Munchausen syndrome. Factitious disorders with all grades of severity are equally distributed between genders. Both factitious disorder and malingering seem to be significantly under- and misdiagnosed. The present study demonstrates that one important obstacle to therapeutic and/or pragmatic interventional studies in unselected series of patients with Munchausen syndrome is that many physicians are insufficiently aware of the condition with its differential diagnosis. Additionally, healthcare providers seem quite regularly to hesitate to put the correct ICD diagnosis on top of the discharge summary. Even with an increased awareness of Munchausen syndrome by health professionals, there remains a rarity of the disorder, necessitating that future systematic, interventional/therapeutic studies, if possible at all, must be performed using a multicenter design on a national basis or even in several countries.

Since, according to our results, the vast majority of patients with the F68.1 diagnosis did not have true Munchausen syndrome, for further studies, there is also a need for subclassification, as also proposed by others [5]. In the ICD, this could be an additional code below F68.1, for example, F68.14.

## Figures and Tables

**Table 1:** Number and age (mean and range) of patients with the diagnosis code F68.1 or Z76.5 in ICD-10 made by Norwegian healthcare providers in the years 2008 to 2016.

		Incidence (number of patients/year/million inhabitants)^∗^
Total F68.1	*n* = 237	5.3
Males	*n* = 138 (58%)	3.1
Age (y)	39 (16–74)	
Females	*n* = 99 (42%)	2.2
Age (y)	37 (8–84)	
Total Z76.5	*n* = 52	1.2
Males	*n* = 40 (77%)	0.9
Age (y)	33 (18–81)	
Females	*n* = 12 (23%)	0.3
Age (y)	43 (16–90)	

^∗^Incidence calculation is based on a total 2012 Norwegian population of 5.0 million.

**Table 2:** Number of patients (female/male) with the diagnosis of F68.1 specified by health sectors.

Somatic	Psychiatric/psychological adults	Psychiatric/psychological children/adolescents	Somatic specialists with operation agreement	Psychiatric/psychological specialists with operation agreement
40/21	31/57	1/0	1/0	26/60

**Table 3:** List of 24 patients with F68.1 diagnosis from health institutions remembered by healthcare providers.

Gender	Age (yr) first-last contact	Number of admission/contacts	Number of institutions	Number of main/additional diagnosis	Corrected/adjusted diagnosis^∗^
F	21-26	17	2	17/0	F68.-Munchausen syndrome
F	35-39	74	7	6/68	F68.-Munchausen syndrome
M	25	10	1	0/10	F68.13
M	27-28	32	2	0/32	F68.13
F	22-24	12	4	6/6	F68.11
F	58	69	2	69/0	F68.11
M	35-36	36	2	6/30	F68.13
F	34	1	1	0/1	F68.11
F	22	4	1	0/1	F68.11
F	31-33	4	1	2/2	F68.13
F	17-18	12	1	12/0	F68.13
F	42	2	1	2/0	Munchausen by proxy
M	36	1	1	1/0	Z76.5
M	55	3	1	0/3	Z76.5
M	36	7	1	6/1	Z76.5
M	31	1	1	1/0	Z76.5
F	32-39	4	4	2/2	Z76.5
M	26	1	1	0/1	Z76.5
F	36-42	88	2	0/88	F71
F	51-52	34	1	0/34	F29.0
M	39-43	6	1	6/0	G20
K	83	1	1	1/0	F41.1
M	35	1	1	0/1	F29
F	52	1	1	1/0	F45.0

^∗^The corrected and/or adjusted diagnosis of the original F68.1 diagnosis code after the physicians or psychologists who made this diagnosis in the first place were contacted and given supplementary information about the diagnostic criteria and the 4 codes below the American 2018 ICD10-CM diagnosis code F68.1.

## Data Availability

The datasets used and/or analyzed during the current study are available from the corresponding author on reasonable request, provided that permission is granted from the Norwegian Patient Registry (NPR).

## Abbreviations

ICD: International classification of diseases
NPR: Norwegian Patient Registry.
